# Prediction Method for Fault-Induced Frequency Response Characteristics in Wind-Integrated Power Systems Using Wide-Area Measurement Data

**DOI:** 10.3390/e27111134

**Published:** 2025-11-02

**Authors:** Yi Hu, Jinglin Luo, Tao Wang, Xiaoqin Lv, Yufei Teng, Xiaopeng Li, Jian Li

**Affiliations:** 1 School of Electrical Engineering and Electronic Information, Xihua University, Chengdu 610039, China; 2 School of Electrical Engineering, Southwest Jiaotong University, Chengdu 611756, China; 3 State Grid Sichuan Electric Power Research Institute, Chengdu 610039, China

**Keywords:** wind-integrated power systems with high penetration, wide-area measurement data, multi-wind-speed scenarios, frequency characteristic prediction, small-signal analysis, information entropy

## Abstract

The decoupling properties and low-inertia characteristics of large-scale wind power have heightened concerns regarding power grid frequency stability, particularly as modern power systems impose stringent frequency regulation requirements on wind integration, leading to an increased complexity of frequency response characteristics under fault conditions. To address this challenge in high-wind-penetration grids, this paper proposes a post-fault frequency dynamics analysis method capable of concurrently accommodating multi-wind-speed scenarios through three key innovations: the linearization of traditional AC system components (including network equations, composite load models, and generator prime mover-governor systems) to establish nodal power increment equations; the development of wind turbine frequency regulation models under diverse wind conditions using small-signal analysis, incorporating regional operational disparities and refined by information entropy-based reliability quantification for adaptive parameter adjustment; and the derivation of the system state equation for post-fault frequency response using wide-area measurement system (WAMS) data, yielding an analytical model that captures region-specific regulation characteristic disparities for physically faithful frequency analysis. Validation via tailored IEEE 39-node simulations convincingly demonstrates the method’s effectiveness and superiority in handling fault-induced transients and wind variability.

## 1. Introduction

With the large-scale grid-off of wind power, the penetration rate of wind power in modern power systems continues to increase. While alleviating the depletion of fossil energy and improving the global ecological environment, the strong randomness of its large-scale output and its own low-inertia characteristics have also brought serious challenges to the frequency stability analysis of the power system.

In conventional power systems dominated by large thermal and hydro units, system inertia is concentrated in synchronous generators. Consequently, the fault-induced frequency response characteristics of the entire system primarily depend on these large units. Wind turbines, however, exhibit low rotational inertia due to their smaller installed capacity and the decoupled relationship between rotor speed and system frequency. Historically, their frequency regulation contribution has been either neglected or oversimplified.

At present, there are two main ways for wind turbines to participate in power system FM. The first is a backup method; that is, the fan runs in a load-reducing state and releases standby power for frequency support when the frequency fluctuates. There are two ways to reduce the load, namely, overspeed reduction [1] and paddle angle reduction [2]. The second is that the fan works in the maximum power point tracking (MPPT) method to provide frequency support by releasing the rotor kinetic energy [3,4].

Due to significant differences in frequency regulation characteristics between wind turbines and conventional thermal/hydro power units, the dynamic frequency behavior of power grids with high wind penetration becomes increasingly complex under fault conditions. For this reason, there have been relevant studies to explore the frequency evolution characteristics of the power grid after large-scale wind power access. Ye H et al. [5] and Zhang J et al. [6] propose an improved system frequency response (SFR) model that takes into account the available inertia and sagging control response of wind farms, which can well simulate the frequency response characteristics of the system under wind power fluctuations. Dai J et al. [7] and Huang H et al. [8] overcome the limitations of traditional methods, fully consider the different operating areas and wind speed disturbances of wind turbines, use the small-signal analysis theory to establish a wind power frequency response model that participates in primary frequency control, and integrate the model into the traditional SFR model to obtain an extended S. The FR model analyzes in detail the impact of initial wind speed, wind speed disturbance and wind power permeability on the frequency response characteristics of the system. Sun M et al. [9] consider the rotor dynamics model of the wind turbine set, the aerodynamic model of the wind turbine, the MPPT control, and the auxiliary frequency control, and construct a system frequency response analysis model of the wind turbine running in MPPT mode. Zhao C et al. [10], in order to effectively evaluate the frequency support ability of wind power generation systems, established an SFR model under the joint action of synchronous generators, wind farms, and low-frequency load-reduction devices, and analyzed the impact of wind farm output power on SFR according to the time domain of the model. Shi Z et al. [11] have established a model for system frequency response composed of a variety of resources, considering the three stages of FM response of wind turbines: the inertial control release rotor kinetic energy stage, the rotor speed recovery stage, and the return-to-MPPT-control stage. By reducing the order of the model, the analytical expression of the lowest frequency point and quasi-steady state are derived, as is the analytic expression of frequency. Huang J et al. [12], on the basis of Ye H et al.’s work [5], handle the linearization error of the SFR model by introducing the quasi-steady-state additional active power output of the wind turbine as an intermediate variable. Zhang X et al. [13] propose a segmented drop-order frequency response (P-ROFR) model. Considering that different frequency response stages have different requirements for the ROFR model, for the full-order model that takes into account the changes in the fan working point, they constructed a high-precision, second-order, drop-order model in the three stages of initial, transient, and steady-state, respectively, and analyzed in detail the relationship between the frequency response index and the auxiliary frequency-controller coefficient.

The above literature approaches the traditional SFR model by building different FM characteristic models of wind turbines. But, in general, there are some limitations: (1) The SFR model is a low-order equivalent model that aggregates and simplifies the power grid. On the one hand, it ignores other dynamic elements, such as the power grid network and load. Regarding the impact on the part of the frequency characteristics, on the other hand, it cannot distinguish the differences in the frequency response characteristics of different types of conventional units and wind turbines. (2) In a high-permeability power grid, wind farms are widely distributed, and there are obvious differences in the operating status and FM characteristics of different wind farms at the same time. A single FM characteristic model cannot truly reflect the change in the overall frequency response of the system. Therefore, the existing frequency response model of wind power systems is only applicable to small-scale, low-permeability wind power grids, and its accuracy and applicability to large-scale power grids with high permeability are obviously insufficient.

To address the challenges of accurate frequency response prediction in high-wind-penetration grids during faults, this paper proposes a linearized frequency dynamic response prediction method driven by wide-area measurement data. Leveraging panoramic grid-state information acquired from pre-fault and post-fault wide-area measurements, the method constructs a predictive model capable of finely characterizing post-disturbance system dynamics, effectively overcoming the limitations of existing frequency response analysis approaches. The primary contributions are as follows:

(1) The system network equations, composite load model, and generator prime mover-governor system are linearized to obtain conventional grid node power increment equations that accurately reflect the impact of network losses and dynamic component characteristics on power balance.

(2) Accounting for diverse wind regimes (regional variations) and control strategies, corresponding wind turbine frequency regulation characteristic models during faults are established using small-signal analysis. Based on information entropy, the frequency regulation reliability of each wind farm is quantified, enabling the simultaneous reflection of frequency response characteristic differences among wind farms during contingencies.

(3) Integrating wide-area measurement data, node power equations, and wind power regulation models, a state-space equation for post-fault system frequency response is constructed. Solving this equation yields a frequency dynamic response analysis model that concurrently accommodates regional wind speed heterogeneity and differentiated frequency regulation characteristics of wind turbines across grid regions. This achieves high-fidelity characterization of frequency stability characteristics in high-wind-penetration power grids during faults.

The rest of this paper is organized as follows: Section 2 derives the node power increment equation for traditional power systems; Section 3 constructs the wind farm node power increment equation considering the frequency modulation effect of wind farms under different wind conditions; Section 4 proposes a frequency dynamic response analysis model considering the differences in frequency modulation characteristics of multiple wind farms; Section 5 provides a simulation analysis example; and finally, Section 6 draws conclusions.

## 2. AC Node Power Increment Equations

### 2.1. System Center of Inertia Frequency

The power system frequency exhibits spatiotemporal distribution characteristics. Following a disturbance, frequencies at various nodes oscillate around the system’s center-of-inertia frequency. Over time, these nodal frequencies converge toward the center-of-inertia frequency.

The incremental equation for the system’s center-of-inertia frequency $\omega_{\mathrm{sys}}$ can be expressed as follows:

(1) $$\Delta \omega_{\mathrm{sys}}=\sum_{i=1}^{n}(H_{i}\Delta \omega_{i})/\sum_{i=1}^{n}H_{i}$$

In the formula, $H_{i}$ represents the inertia time constant of the *i*-th generator in the power grid; $\Delta \omega_{i}$ represents the angular frequency increment of the *i*-th generator in the power grid; n represents the number of generator nodes in the power grid after disturbance.

### 2.2. Generator Node Power Increment Equation

The generator adopts a classical second-order model, and the rotor motion equation is linearized, resulting in the generator node power increment equation as follows:

(2) $$\left\{\begin{array}{l} \frac{d\Delta \theta_{i}}{dt}=(\Delta \omega_{i}-\Delta \omega_{k})\cdot \omega_{B}\\ M_{i}\frac{d\Delta \omega_{i}}{dt}=\Delta P_{mi}-\Delta P_{eGi}+P_{ai0} \end{array}\right.$$

In the formula, $\theta_{i}$ represents the power angle of the *i*-th generator; $\Delta P_{mi}$, $\Delta P_{ei}$ represent the system reference frequency, and represent the mechanical power increment and electromagnetic power increment on the rotor of the *i*-th generator; $M_{i}$ represents the inertia constant of the *i*-th generator.

### 2.3. Prime Mover-Governor Power Increment Equation

The prime mover-governor affects the rotor’s motion by altering its mechanical output, thereby modifying the frequency dynamics and active power of the power system. In this paper, the second-order TGOV1 model is selected to describe the dynamic characteristics of the prime mover-governor system:

(3) $$\left\{\begin{array}{l} \Delta {\overset{\cdot }{k}}_{i}=-\frac{1}{T_{1i}}\cdot \Delta k_{i}-\frac{1}{R_{i}T_{1i}}\Delta \omega_{i}\\ \Delta {\overset{\cdot }{P}}_{Ti}=\frac{1}{T_{3i}}\Delta k_{i}+\frac{T_{2i}}{T_{3i}}\cdot \Delta {\overset{\cdot }{k}}_{i}-\frac{1}{T_{3i}}\Delta P_{Ti}\\ \Delta P_{mi}=\Delta P_{Ti}-D_{ti}\cdot \Delta \omega_{i} \end{array}\right.$$

In the formula, $\Delta P_{Ti}$ represents the increment of turbine power; $\Delta k_{i}$ represents the increment of turbine valve opening; $T_{1i}$, $T_{2i}$, $T_{3i}$ represent the turbine-governor time parameters; $R_{i}$ represents the turbine-governor adjustment coefficient; $\Delta P_{mi}$ represents the increment of turbine output mechanical power; $D_{ti}$ represents the turbine-governor damping coefficient.

From Equation (3), we can derive the prime mover-governor state equation as follows:

(4) $$\left[\begin{array}{c} \Delta {\dot{P}}_{Ti}\\ \Delta {\dot{k}}_{i} \end{array}\right]=\left[\begin{array}{cc} -\frac{1}{T_{3i}} & \frac{T_{1i}-T_{2i}}{T_{1i}T_{3i}}\\ 0 & -\frac{1}{T_{1i}} \end{array}\right]\left[\begin{array}{c} \Delta P_{Ti}\\ \Delta k_{i} \end{array}\right]-\left[\begin{array}{c} \frac{T_{2i}}{R_{i}T_{1i}T_{3i}}\\ \frac{1}{R_{i}T_{1i}} \end{array}\right]\Delta \omega_{i}$$

(5) $$\Delta P_{mi}=\left[\begin{array}{cc} 1 & 0 \end{array}\right]\left[\begin{array}{c} \Delta P_{Ti}\\ \Delta k_{i} \end{array}\right]-D_{ti}\Delta \omega_{i}$$

### 2.4. Load Node Power Increment Equation

Taking into account the voltage and frequency variation effects of the load, the power injected into the load node can be expressed as:

(6) $$\left\{\begin{array}{l} P_{li}=-P_{li0}[a_{pi}{\left(\frac{U_{i}}{U_{i0}}\right)}^{2}+b_{pi}(\frac{U_{i}}{U_{i0}})+c_{pi}](1+{\left.\frac{dP_{0}}{d\omega_{0}}\right|}_{\omega_{0}}\Delta \omega_{0})\\ Q_{li}=-Q_{1i0}[a_{qi}{\left(\frac{U_{i}}{U_{i0}}\right)}^{2}+b_{qi}(\frac{U_{i}}{U_{i0}})+c_{qi}](1+{\left.\frac{dP_{0}}{d\omega_{0}}\right|}_{\omega_{0}}\Delta \omega_{0}) \end{array}\right.$$

In the formula, $P_{li0}$, $Q_{li0}$ represent the initial active power and reactive power of the node load, respectively; $a_{pi}$, $b_{pi}$, $c_{pi}$ are the constant power, constant current, and constant impedance proportional coefficients of the active load, respectively; and $a_{qi}$, $b_{qi}$, $c_{qi}$ are the constant power, constant current, and constant impedance proportional coefficients of the reactive load, respectively. They satisfy the constraint relationships of $a_{pi}$ + $b_{pi}$ + $c_{pi}$ = 1 and $a_{qi}$ + $b_{qi}$ + $c_{qi}$ = 1 to ensure the completeness of the overall load characteristic description.

By linearizing Equation (6), we obtain the incremental equation for the injection power at the load node as follows:

(7) $$\left\{\begin{array}{l} \Delta P_{Li}=-\frac{\partial P_{li}}{\partial \omega_{i}}{|}_{0^{+}}\Delta \omega_{i}-\frac{\partial P_{li}}{\partial V_{Li}/V_{Li}}{|}_{0^{+}}\frac{\Delta V_{Li}}{V_{Li}}\\ \Delta Q_{Li}=-\frac{\partial Q_{li}}{\partial \omega_{i}}{|}_{0^{+}}\Delta \omega_{i}-\frac{\partial Q_{li}}{\partial V_{Li}/V_{Li}}{|}_{0^{+}}\frac{\Delta V_{Li}}{V_{Li}} \end{array}\right.$$

### 2.5. System Node Power Increment Equations

At the critical post-fault instant *t* = 0^+^, the node-injected power equations are as follows:

(8) $$P_{i0^{+}}=V_{i0^{+}}\sum_{j=1}^{n}V_{j0^{+}}(G_{ij}\cos\theta_{ij0^{+}}+B_{ij}\sin\theta_{ij0^{+}})$$

(9) $$Q_{i0^{+}}=V_{i0^{+}}\sum_{j=1}^{n}V_{j0^{+}}(G_{ij}\sin\theta_{ij0^{+}}-B_{ij}\cos\theta_{ij0^{+}})$$

In the formula, $P_{i0^{+}}$, $Q_{i0^{+}}$ represent the active power and reactive power injected into the nodes, respectively; $V_{i0^{+}}$, $V_{j0^{+}}$ represent the voltage amplitude of the nodes, respectively; $G_{ij}$, $B_{ij}$ represent the mutual admittance between nodes; $\theta_{ij0^{+}}$ represents the phase angle difference in node voltage.

Linearizing (8) and (9) yields the post-fault system node power increment equation:

(10) $$\left[\begin{array}{l} \Delta P_{G}\\ \Delta P_{L}\\ \Delta Q_{L} \end{array}\right]=-\left[\begin{array}{ccc} H_{\mathrm{GG}} & H_{\mathrm{GL}} & N_{G}\\ H_{\mathrm{LG}} & H_{\mathrm{LL}} & N_{L}\\ J_{\mathrm{LG}} & J_{\mathrm{LL}} & L_{L} \end{array}\right]\left[\begin{array}{l} \Delta \theta_{G}\\ \Delta \theta_{L}\\ U_{L}^{-1}\Delta U_{L0^{+}} \end{array}\right]$$

In the formula, $\Delta P_{G}$ represents the electromagnetic power increment of the generator node; $\Delta P_{L}$, $\Delta Q_{L}$, respectively, represent the active and reactive power increments of the load node; $\Delta \theta_{G}$, $\Delta \theta_{L}$, respectively, represent the phase angle increments of the generator and load nodes; $\Delta V_{L}$ represents the voltage amplitude change in the load node; $V_{L0^{+}}$ represents the voltage amplitude of load nodes at the critical post-fault instant $t=0^{+}$; the expressions for H, N, J, L are the same as the expressions for the elements of the Jacobian matrix in the Newton–Raphson power flow calculation, obtained from wide-area measurement data capturing nodal voltages and phase angles at $t=0^{+}$ immediately following the fault.

## 3. Wind Power Node Power Increment Equations

Currently, wind turbine generators generally achieve load shedding through the addition of virtual inertia integrated control, overspeed control, pitch angle control, and other methods. Different control methods produce significant differences in load shedding and frequency modulation effects. Considering the large number and wide distribution of wind farms in high-wind-power-penetration grids, wind farms in different regional locations will face different wind speed conditions. Therefore, under different wind speed conditions, wind turbine generators will adopt different control methods, operate in different states, and produce different frequency modulation effects on the system. Therefore, for high-wind-power-penetration grids, the differences in frequency modulation characteristics of wind turbine groups due to their spatial dispersion cannot be ignored. It is essential to establish a frequency response analysis model for power systems under fault conditions that concurrently considers diverse wind speed conditions and varying wind turbine frequency regulation contributions.

### 3.1. Frequency Response Mathematical Model for Low Wind Speed

When the wind farm operates under low-wind-speed conditions, the rotational reserve of the wind turbine is relatively small. To ensure the stability of the wind turbine generator, the rotational reserve can be disregarded. According to reference [7], at this time, the rotor speed is relatively low, and the pitch angle does not need to act, so the influence factor of the pitch angle is not considered. The mechanical torque can be expressed as follows:

(11) $$\Delta T_{m}(s)=(\frac{K_{w}K_{c}v^{3}\lambda }{w^{2}}-\frac{K_{w}v^{3}C_{p}}{w^{2}})\Delta w(s)+(\frac{3K_{w}C_{p}v^{2}}{w}-\frac{K_{w}K_{c}v^{2}\lambda }{w})\Delta v(s)$$

In the formula, $\Delta T_{m}(s)$ is the mechanical power torque increment; v is the wind speed; λ is the tip speed ratio; $C_{pref}$ is the reference value of the wind energy utilization coefficient; $K_{w}$ is the wind energy-capture ratio factor.

Synthetic inertial control is inactive during low-wind-speed-operation. Hence, the change in electromagnetic power during faults is solely induced by wind speed variations:

(12) $$\Delta T_{e}(s)=2K_{w}w\Delta w(s)$$

According to the rotor motion equation of the fan,

(13) $$\Delta T_{e}(s)=2K_{w}w\Delta w(s)$$

can obtain

(14) $$\Delta w(s)=\frac{K_{w}v^{2}w(3C_{p}+K_{c}\lambda )}{K_{w}v^{3}(K_{c}\lambda -C_{p})-2K_{p}w^{3}-2K_{c}H_{w}sw^{2}}\Delta v(s)$$

When running at low wind speed, according to the electromagnetic power torque and Formula (13) of the fan, it can be obtained that the output electromagnetic power increment of the fan at this time is as follows:

(15) $$\Delta P_{\mathrm{ew}1}(s)=\frac{a_{l}}{b_{l}-c_{l}s}\Delta v(s)$$

among

(16) $$a_{l}=2K_{w}^{2}v^{2}w^{2}(3C_{p}+K_{c}\lambda )$$

(17) $$b_{l}=K_{w}v^{3}(K_{c}\lambda -C_{p})-2K_{w}w^{3}$$

(18) $$c_{l}=2K_{c}H_{w}sw^{2}$$

For low-wind-speed operation, applying the inverse Laplace transform and incorporating parameters at the fault inception operating point yields the state equation of the wind turbine:

(19) $$\Delta {\dot{P}}_{\mathrm{ew}1}=\frac{b_{l}}{c_{l}}\Delta P_{\mathrm{ew}1}-\frac{a_{l}}{c_{l}}\Delta v_{l}$$

### 3.2. Frequency Response Mathematical Model for Medium Wind Speed

When wind farms operate under medium-wind-speed conditions, wind turbines possess sufficient rotational margin, enabling the utilization of rotor kinetic energy to respond to frequency deviations during faults. A combined control strategy integrating overspeed control and pitch angle adjustment is adopted. When the output power is below the rated value, the pitch angle remains near 0 degrees, and rotor speed regulation adjusts the output power. At higher wind speeds where rotor speed is elevated, the pitch angle is increased to shift the operating point from the maximum power point to a stable deloaded point, reserving power support capability.

As indicated in Reference [7], during fault events, the electromagnetic power increment comprises contributions from synthetic inertial control and overspeed deloading reserves. The electromagnetic power increment from synthetic inertial control is as follows:

(20) $$\Delta T_{e\_\mathrm{VIC}}=\frac{-k_{d}\frac{d\Delta f}{dt}-k_{p}\Delta f}{w}=-\frac{k_{d}s+k_{p}}{w}\Delta f(s)$$

In the formula, $k_{d}$ is the virtual inertial coefficient and $k_{p}$ is the sagging coefficient.

The residual electromagnetic power increment of overspeed load reduction is as follows:

(21) $$\Delta T_{e\text{\_}\mathrm{del}}=\frac{\partial T_{e}}{\partial \omega }\Delta w=[2(1-K_{\mathrm{del}})K_{\mathrm{opt}}w+2K_{\mathrm{del}}K_{\mathrm{opt}}\frac{w_{2}-w}{w_{2}-w_{1}}-\frac{K_{\mathrm{del}}K_{\mathrm{opt}}w^{3}}{\omega_{2}-\omega_{1}}]\Delta w$$

Among the terms, $w_{1}$, $w_{2}$ are the angular velocity of the fan at maximum power and load-reduction power, respectively. The total electromagnetic power increment $\Delta T_{e}$ is expressed as follows:

(22) $$\Delta T_{e}=-\frac{k_{d}s+k_{p}}{w}\Delta f(s)+[2(1-K_{\mathrm{del}})K_{\mathrm{opt}}w+2K_{\mathrm{del}}K_{\mathrm{opt}}\frac{w_{2}-w}{w_{2}-w_{1}}-\frac{K_{\mathrm{del}}K_{\mathrm{opt}}w^{3}}{\omega_{2}-\omega_{1}}]\Delta w$$

Substitute Formulas (11), (13) and (22) to obtain the following:

(23) $$\Delta P_{\mathrm{ew}2}(s)=V_{m}(s)\Delta v(s)+G_{m}(s)\Delta f(s)$$

(24) $$V_{m}(s)=\frac{a_{m}}{b_{m}s+1}$$

(25) $$G_{m}(s)=-\frac{c_{m}s^{2}+d_{m}s+e_{m}}{b_{m}s+1}$$

among

(26) $$\begin{array}{l} a_{m}=\frac{\left[3(1-K_{del})K_{w}w^{2}+3K_{del}K_{w}w^{2}\frac{\left(w_{del}-w\right)}{\left(w_{del}-w_{\max}\right)}-\frac{K_{del}K_{w}w^{3}}{\left(w_{del}-w_{\max}\right)}][3K_{w}wv^{2}C_{pref}-K_{w}K_{c}wv^{2}\lambda \right]}{2(1-K_{del})K_{w}w^{3}+K_{w}v^{3}C_{pref}+2K_{del}K_{w}w^{3}\frac{\left(w_{del}-w\right)}{\left(w_{del}-w_{\max}\right)}-\frac{K_{del}K_{w}w^{4}}{\left(w_{del}-w_{\max}\right)}-K_{w}K_{c}wv^{2}}\\ b_{m}=\frac{2K_{M}H_{w}w^{2}}{2(1-K_{del})K_{w}w^{3}+K_{w}v^{3}C_{pref}+2K_{del}K_{w}w^{3}\frac{\left(w_{del}-w\right)}{\left(w_{del}-w_{\max}\right)}-\frac{K_{del}K_{w}w^{4}}{\left(w_{del}-w_{\max}\right)}-K_{w}K_{c}wv^{2}}\\ c_{m}=\frac{2K_{M}H_{w}K_{v}w^{2}}{2(1-K_{del})K_{w}w^{3}+K_{w}v^{3}C_{pref}+2K_{del}K_{w}w^{3}\frac{\left(w_{del}-w\right)}{\left(w_{del}-w_{\max}\right)}-\frac{K_{del}K_{w}w^{4}}{\left(w_{del}-w_{\max}\right)}-K_{w}K_{c}wv^{2}}\\ d_{m}=\frac{2K_{M}H_{w}w^{2}/R_{v}+K_{v}K_{p}v^{3}C_{pref}-(1-K_{del})K_{v}K_{w}w^{3}-K_{del}K_{w}K_{v}w^{3}\frac{\left(w_{del}-w\right)}{\left(w_{del}-w_{\max}\right)}-K_{del}K_{w}K_{v}w^{2}}{2(1-K_{del})K_{w}w^{3}+K_{w}v^{3}C_{pref}+2K_{del}K_{w}w^{3}\frac{\left(w_{del}-w\right)}{\left(w_{del}-w_{\max}\right)}-\frac{K_{del}K_{w}w^{4}}{\left(w_{del}-w_{\max}\right)}-K_{w}K_{c}wv^{2}}\\ e_{m}=\frac{K_{w}K_{M}H_{w}v^{3}C_{pref}/R_{v}-(1-K_{del})K_{w}w^{3}/R_{v}-K_{w}K_{v}wv^{2}/R_{v}-K_{del}K_{w}K_{v}w^{2}\frac{\left(w_{del}-w\right)}{\left(w_{del}-w_{\max}\right)}}{2(1-K_{del})K_{w}w^{3}+K_{w}v^{3}C_{pref}+2K_{del}K_{w}w^{3}\frac{\left(w_{del}-w\right)}{\left(w_{del}-w_{\max}\right)}-\frac{K_{del}K_{w}w^{4}}{\left(w_{del}-w_{\max}\right)}-K_{w}K_{c}wv^{2}} \end{array}$$

For medium-wind-speed operation, applying the inverse Laplace transform and incorporating parameters at the fault inception operating point yields the state equation of the wind turbine:

(27) $$\Delta {\dot{P}}_{\mathrm{ew}2}=-\frac{1}{b_{m}}\Delta P_{\mathrm{ew}2}+\frac{a_{m}}{b_{m}}\Delta v_{m}+\frac{c_{m}}{b_{m}}\Delta \ddot{f}+\frac{d_{m}}{b_{m}}\Delta \dot{f}+\frac{e_{m}}{b_{m}}\Delta f$$

### 3.3. Frequency Response Mathematical Model for High Wind Speed

When wind farms operate under high-wind-speed conditions, the rotor speed of wind turbines approaches its rated value. Adjusting output power by varying rotor speed becomes impractical. Therefore, pitch angle control is employed to acquire frequency regulation reserves. By modifying the blade pitch angle, the mechanical energy captured by the wind turbine is adjusted, maintaining operation below the maximum power point to reserve power for supporting potential power deficits caused by system faults.

According to reference [7], during fault events, the wind turbine’s electromagnetic power increment arises from the synthetic inertial control and pitch angle action, expressed as follows:

(28) $$\Delta T_{e}=-\frac{k_{d}s+k_{p}}{w}\Delta f(s)+2K_{\mathrm{opt}}w\Delta w$$

Substitute Formulas (11), (13) and (28) to obtain the following:

(29) $$\Delta P_{\mathrm{ew}3}(s)=V_{h}(s)\Delta v(s)+G_{h}(s)\Delta f(s)$$

(30) $$V_{h}(s)=\frac{a_{h}}{b_{h}s+1}$$

(31) $$G_{h}(s)=-\frac{c_{h}s^{2}+d_{h}s+e_{h}}{b_{h}s+1}$$

(32) $$\begin{array}{l} a_{h}=\frac{2K_{H}H_{w}w^{2}}{2K_{w}w^{3}+K_{w}v^{3}C_{pref}-K_{w}K_{c}wv^{2}}\\ b_{h}=\frac{3K_{H}H_{w}w^{2}(3C_{pref}-K_{c}\lambda_{ref})}{2K_{w}w^{3}+K_{w}v^{3}C_{pref}-K_{w}K_{c}wv^{2}}\\ c_{h}=\frac{3K_{H}H_{w}w^{2}K_{v}}{2K_{w}w^{3}+K_{w}v^{3}C_{pref}-K_{w}K_{c}wv^{2}}\\ d_{h}=\frac{3K_{H}H_{w}w^{2}1/R_{v}-3K_{H}K_{w}K_{v}w^{2}+2K_{v}K_{w}w^{3}-K_{v}K_{P}wv^{2}}{2K_{w}w^{3}+K_{w}v^{3}C_{pref}-K_{w}K_{c}wv^{2}}\\ e_{h}=\frac{1/R_{v}(2K_{H}K_{w}w^{3}-2K_{v}K_{w}w^{3}+K_{H}K_{w}v^{3}C_{pref})-3w^{2}1/R_{v}}{2K_{w}w^{3}+K_{w}v^{3}C_{pref}-K_{w}K_{c}wv^{2}} \end{array}$$

For high-wind-speed operation, applying the inverse Laplace transform and incorporating parameters at the fault inception operating point yields the state equation of the wind turbine:

(33) $$\Delta {\dot{P}}_{\mathrm{ew}3}=-\frac{1}{b_{m}}\Delta P_{\mathrm{ew}3}+\frac{a_{m}}{b_{m}}\Delta v_{h}+\frac{c_{m}}{b_{m}}\Delta \ddot{f}+\frac{d_{m}}{b_{m}}\Delta \dot{f}+\frac{e_{m}}{b_{m}}\Delta f$$

### 3.4. Frequency Regulation Parameter Modification Based on Information Entropy

The frequency regulation capability of wind turbine units is fundamentally characterized by the reliability of their active power dynamic response. It should be noted that the inherent stochasticity of wind power output leads to significant differences in historical operational stability among wind farms within the same theoretical wind speed range. These differences directly impact the credibility of power support during faults; i.e., wind farms with high historical volatility inherently exhibit weaker frequency regulation capabilities than stably operated counterparts of the same type.

To quantify this reliability difference, this paper introduces information entropy as a metric. Based on historical wide-area measurement system data, the probability distribution of equivalent wind speed sequences for each wind farm is calculated, and its information entropy $H_{j}$ is derived as follows:

(34) $$H_{j}=-\sum_{1}^{N_{b}}P(v_{i})\log_{2}P(v_{i})$$

In the formula, $H_{j}$ denotes the historical equivalent wind speed entropy value of the j-th wind farm; $N_{b}$ is the number of wind speed intervals; $P(v_{i})$ is the probability of wind speed falling within the i-th interval.

A higher entropy value $H_{j}$ indicates greater instability in the wind farm’s historical operation and lower reliability of its current frequency regulation capability. Accordingly, an entropy weighting factor $\alpha_{j}$ is constructed to correct the reliability of the wind farm’s frequency regulation parameters:

(35) $$\alpha_{j}=e^{-\gamma {\tilde{H}}_{j}}$$

In the formula, ${\tilde{H}}_{j}$ is the normalized entropy value and γ is a regulation coefficient greater than zero, controlling the sensitivity to entropy influence. Here, $\alpha_{j}\in (0,1]$, with values closer to 1 indicating higher frequency regulation reliability of the wind farm.

During low-wind-speed operation, wind turbine units possess minimal rotational reserves. Their weak frequency regulation capability primarily manifests in the natural response of electromagnetic power to wind speed fluctuations. This response process is closely related to the wind energy capture proportionality factor $K_{c}$. Therefore, this parameter should undergo entropy weighting correction to reflect the reliability of its response capability:

(36) $$K_{c,j}^{eff}=\alpha_{j}\cdot K_{c,j}$$

In the formula, $K_{c,j}$ denotes the original proportionality factor for the j-th wind farm in the low-wind-speed model, and $K_{c,j}^{eff}$ represents the effective value after reliability correction.

Conversely, during high-wind-speed operation, wind turbine units provide frequency regulation reserves through pitch angle control. Their frequency regulation capability is dominated by the response strength of the pitch angle-control coefficient $K_{\beta }$ to frequency deviations. Hence, this parameter requires entropy weighting correction:

(37) $$K_{\beta ,j}^{eff}=\alpha_{j}\cdot K_{\beta ,j}$$

In the formula, $K_{\beta ,j}$ is the original pitch angle-control coefficient for the j-th wind farm in the high-wind-speed model, and $K_{\beta ,j}^{eff}$ is the reliability-corrected effective value.

**Figure 1:**
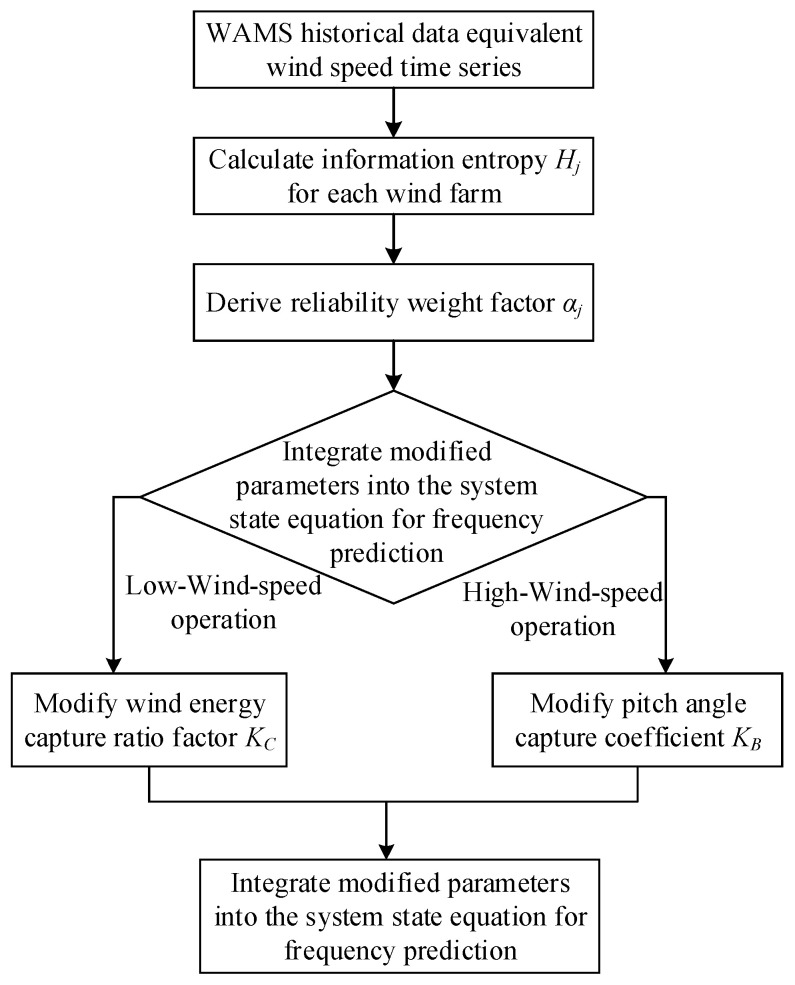
Information Entropy Method Flow Chart.

Ultimately, regardless of the operational conditions encountered, the key parameters within wind farm frequency regulation models must undergo weighting correction based on their respective historical operational entropy values $\alpha_{j}$. Consequently, when integrating the state equations describing wind turbine units under low-wind-speed, medium-wind-speed, and high-wind-speed operating regimes—as detailed in Equations (19), (27), and (33)—into the system-level state equation given by Equation (47), all parameters must adopt the corrected effective values $\left(K_{c,j}^{eff},K_{w,j}^{eff},K_{v,j}^{eff},K_{\beta ,j}^{eff}\right)$. The method flow is shown in Figure 1 below:

## 4. Frequency Dynamics Prediction Model Considering Wind Power Regulation Characteristics

In order to distinguish between traditional generator nodes and wind power nodes under different working conditions, the power increment equation of the system node shown in Formula (15) can be extended to the following:

(38) $$\left[\begin{array}{c} \Delta P_{\mathrm{eG}}\\ \Delta P_{\mathrm{eW}1}\\ \Delta P_{\mathrm{eW}2}\\ \Delta P_{\mathrm{eW}3}\\ \Delta P_{L}\\ \Delta Q_{L} \end{array}\right]=-\left[\begin{array}{cccccc} H_{\mathrm{GG}} & H_{G1} & H_{G2} & H_{G3} & H_{\mathrm{GL}} & N_{G}\\ H_{1G} & H_{11} & H_{12} & H_{13} & H_{1L} & N_{1}\\ H_{2G} & H_{21} & H_{22} & H_{23} & H_{2L} & N_{2}\\ H_{3G} & H_{31} & H_{32} & H_{33} & H_{3L} & N_{3}\\ H_{\mathrm{LG}} & H_{L1} & H_{L2} & H_{L3} & H_{\mathrm{LL}} & N_{G}\\ J_{\mathrm{LG}} & J_{L1} & J_{L2} & J_{L3} & J_{\mathrm{LL}} & L_{L} \end{array}\right]\left[\begin{array}{c} \Delta \theta_{G}\\ \Delta \theta_{W1}\\ \Delta \theta_{W2}\\ \Delta \theta_{W3}\\ \Delta \theta_{L}\\ \Delta V_{L}/V_{L0^{+}} \end{array}\right]$$

In order to consider the impact of the load frequency-change effect, use the inertial center frequency of the system to approximate the frequency of each load node, inject the load node into the power increment using Equation (7) and the substitution found in Formula (38), eliminate the active and reactive increments of the load node, and obtain the electromagnetic power increment equation of the generator node as follows:

(39) $$\left[\begin{array}{c} \Delta P_{\mathrm{eG}}\\ \Delta P_{\mathrm{eW}1}\\ \Delta P_{\mathrm{eW}2}\\ \Delta P_{\mathrm{eW}3}\\ 0\\ 0 \end{array}\right]=-\left[\begin{array}{ccccccc} H_{\mathrm{GG}} & H_{G1} & H_{G2} & H_{G3} & H_{\mathrm{GL}} & N_{G} & S_{1}\\ H_{1G} & H_{11} & H_{12} & H_{13} & H_{1L} & N_{1} & S_{2}\\ H_{2G} & H_{21} & H_{22} & H_{23} & H_{2L} & N_{2} & S_{3}\\ H_{3G} & H_{31} & H_{32} & H_{33} & H_{3L} & N_{3} & S_{4}\\ H_{\mathrm{LG}} & H_{L1} & H_{L2} & H_{L3} & H_{\mathrm{LL}} & N^{\prime } & S_{5}\\ J_{\mathrm{LG}} & J_{L1} & J_{L2} & J_{L3} & J_{\mathrm{LL}} & L^{\prime } & S_{6} \end{array}\right]\left[\begin{array}{c} \Delta \theta_{G}\\ \Delta \theta_{W1}\\ \Delta \theta_{W2}\\ \Delta \theta_{W3}\\ \Delta \theta_{L}\\ \Delta V_{L}/V_{L0^{+}}\\ \Delta \omega \end{array}\right]$$

In the formula, $S_{1}=\partial P_{\mathrm{eG}}/\partial \omega$; $S_{2}=\partial P_{\mathrm{eW}1}/\partial \omega$; $S_{3}=\partial P_{\mathrm{eW}2}/\partial \omega$; $S_{4}=\partial P_{\mathrm{eW}3}/\partial \omega$; $S_{5}=\partial P_{L}/\partial \omega$; $S_{6}=\partial Q_{L}/\partial \omega$; $N_{L}^{\prime }=N_{L}-\frac{\partial P_{L}}{\partial V_{L}/V_{L0^{+}}}$; $L_{L}^{\prime }=L_{L}-\frac{\partial Q_{L}}{\partial V_{L}/V_{L0^{+}}}$.

The following can be obtained from the above formula:

(40) $$\left[\begin{array}{c} -\Delta P_{\mathrm{eW}1}-H_{G1}\Delta \theta_{G}-S_{2}\Delta \omega \\ -\Delta P_{\mathrm{eW}2}-H_{G2}\Delta \theta_{G}-S_{3}\Delta \omega \\ -\Delta P_{\mathrm{eW}3}-H_{G3}\Delta \theta_{G}-S_{4}\Delta \omega \\ -H_{\mathrm{LG}}-S_{5}\Delta \omega \\ -J_{\mathrm{LG}}-S_{6}\Delta \omega \end{array}\right]=\left[\begin{array}{ccccc} H_{11} & H_{12} & H_{13} & H_{1L} & N_{1}\\ H_{21} & H_{22} & H_{23} & H_{2L} & N_{2}\\ H_{31} & H_{32} & H_{33} & H_{3L} & N_{3}\\ H_{L1} & H_{L2} & H_{L3} & H_{\mathrm{LL}} & N^{\prime }\\ J_{L1} & J_{L2} & J_{L3} & J_{\mathrm{LL}} & L^{\prime } \end{array}\right]\left[\begin{array}{c} \Delta \theta_{W1}\\ \Delta \theta_{W2}\\ \Delta \theta_{W3}\\ \Delta \theta_{L}\\ \Delta V_{L}/V_{L0^{+}} \end{array}\right]$$

At the same time,

(41) $$-\Delta P_{\mathrm{eG}}-H_{\mathrm{GG}}\Delta \theta_{G}-S_{1}\Delta \omega =\left[\begin{array}{ccccc} H_{G1} & H_{G2} & H_{G3} & H_{\mathrm{GL}} & N_{G} \end{array}\right]\left[\begin{array}{c} \Delta \theta_{W1}\\ \Delta \theta_{W2}\\ \Delta \theta_{W3}\\ \Delta \theta_{L}\\ \Delta V_{L}/V_{L0^{+}} \end{array}\right]$$

ordered as

(42) $$Z={\left[\begin{array}{ccccc} H_{11} & H_{12} & H_{13} & H_{1L} & N_{1}\\ H_{21} & H_{22} & H_{23} & H_{2L} & N_{2}\\ H_{31} & H_{32} & H_{33} & H_{3L} & N_{3}\\ H_{L1} & H_{L2} & H_{L3} & H_{LL} & N^{\prime }\\ J_{L1} & J_{L2} & J_{L3} & J_{LL} & L^{\prime } \end{array}\right]}^{-1}$$

Then you can obtain

(43) $$-\Delta P_{\mathrm{eG}}-H_{GG}\Delta \theta_{G}-S_{1}\Delta \omega =\left[\begin{array}{ccccc} H_{G1} & H_{G2} & H_{G3} & H_{GL} & N_{G} \end{array}\right]\cdot Z\left[\begin{array}{c} -\Delta P_{\mathrm{eW}1}-H_{G1}\Delta \theta_{G}-S_{2}\Delta \omega \\ -\Delta P_{\mathrm{eW}2}-H_{G2}\Delta \theta_{G}-S_{3}\Delta \omega \\ -\Delta P_{\mathrm{eW}3}-H_{G3}\Delta \theta_{G}-S_{4}\Delta \omega \\ -H_{LG}-S_{5}\Delta \omega \\ -J_{LG}-S_{6}\Delta \omega \end{array}\right]$$

ordered as

(44) $$\left[\begin{array}{ccccc} E_{1} & E_{2} & E_{3} & E_{4} & E_{5} \end{array}\right]=\left[\begin{array}{ccccc} H_{G1} & H_{G2} & H_{G3} & H_{GL} & N_{G} \end{array}\right]Z$$

According to Equation (39) for the electromagnetic power increment of the generator node, the voltage phase increment and amplitude increment of the load node can be eliminated:

(45) $$\Delta P_{\mathrm{eG}}=E_{1}\Delta P_{\mathrm{eW}1}+E_{2}\Delta P_{\mathrm{eW}2}+E_{3}\Delta P_{\mathrm{eW}3}+F_{1}\Delta \theta_{G}+F_{2}\Delta \omega +F_{3}$$

among the following relationships, $F_{1}=-H_{GG}+E_{1}H_{G1}+E_{2}H_{G2}+E_{3}H_{G3}$, $F_{2}=-S_{1}+E_{1}S_{2}+E_{2}S_{3}+E_{3}S_{4}+E_{4}S_{5}+E_{5}S_{6}$, $F_{3}=E_{4}H_{LG}+E_{5}J_{LG}$.

Substitute Formulas (5) and (45) into Formula (2), and you can obtain the following:

(46) $$\Delta \dot{\omega }=\Delta P_{T}-\mathrm{diag}\left\{D_{ti}\right\}\Delta \omega -E_{1}\Delta P_{\mathrm{eW}1}-E_{2}\Delta P_{\mathrm{eW}2}-E_{3}\Delta P_{\mathrm{eW}3}-F_{1}\Delta \theta_{G}-F_{2}\Delta \omega +F_{3}$$

Combining Equations (2), (4), (19), (27), (33), and (46), the system state equation characterizing the post-fault frequency dynamic response of high-penetration wind power grids is derived:

(47) $$\left\{\begin{array}{l} \Delta \dot{\theta }=\omega_{B}M_{1}\Delta \omega \\ \Delta \dot{\omega }=\Delta P_{T}-M_{2}\Delta \omega -E_{1}\Delta P_{eW1}-E_{2}\Delta P_{eW2}\\ -E_{3}\Delta P_{eW3}-F_{1}\Delta \theta_{G}-F_{2}\Delta \omega +F_{3}\\ \Delta {\dot{P}}_{T}=M_{3}\Delta k-M_{4}\Delta \omega -M_{5}\Delta P_{T}\\ \Delta \dot{k}=M_{6}\Delta k-M_{7}\Delta \omega \\ \Delta {\dot{P}}_{eW1}=M_{8}\Delta P_{eW1}-M_{9}\Delta v_{1}\\ \Delta {\dot{P}}_{eW2}=-M_{10}\Delta P_{eW2}+M_{11}\Delta \ddot{\omega }+M_{12}\Delta \dot{\omega }+M_{13}\Delta \omega -M_{14}\Delta v_{2}\\ \Delta {\dot{P}}_{eW3}=-M_{15}\Delta P_{eW3}+M_{16}\Delta \ddot{\omega }+M_{17}\Delta \dot{\omega }+M_{18}\Delta \omega -M_{19}\Delta v_{3} \end{array}\right.$$

For the matrices N and M, see Appendix A.

For wind turbines running at medium wind speed and high wind speed, the equation of state can be converted into the following:

(48) $$\left\{\begin{array}{l} \Delta {\dot{P}}_{\mathrm{eW}2}=N_{1}\Delta \theta_{G}+N_{2}\Delta \omega +N_{3}\Delta P_{T}+N_{4}\Delta k+N_{5}\Delta P_{\mathrm{eW}1}\\ +N_{6}\Delta P_{\mathrm{eW}2}+N_{7}\Delta P_{\mathrm{eW}3}+N_{8}-N_{9}\Delta {\dot{P}}_{\mathrm{eW}3}\\ \Delta {\dot{P}}_{\mathrm{eW}3}=N_{10}\Delta \theta_{G}+N_{11}\Delta \omega +N_{12}\Delta P_{T}+N_{13}\Delta k\\ +N_{14}\Delta P_{\mathrm{eW}1}+N_{15}\Delta P_{\mathrm{eW}2}+N_{16}\Delta P_{\mathrm{eW}3}+N_{17}-N_{18}\Delta {\dot{P}}_{\mathrm{eW}2} \end{array}\right.$$

A further derivation can be obtained:

(49) $$\left\{\begin{array}{l} \Delta {\dot{P}}_{\mathrm{eW}2}={\left(N_{9}N_{18}-E\right)}^{-1}[\left(N_{10}N_{9}-N_{1}\right)\Delta \theta_{G}+\left(N_{11}N_{9}-N_{2}\right)\Delta \omega \\ +\left(N_{12}N_{9}-N_{3}\right)\Delta P_{T}+\left(N_{13}N_{9}-N_{4}\right)\Delta k+\left(N_{14}N_{9}-N_{5}\right)\Delta P_{\mathrm{eW}1}\\ +\left(N_{15}N_{9}-N_{6}\right)\Delta P_{\mathrm{eW}2}+\left(N_{16}N_{9}-N_{7}\right)\Delta P_{\mathrm{eW}3}+\left(N_{17}N_{9}-N_{8}\right)]\\ \Delta {\dot{P}}_{\mathrm{eW}3}={\left(N_{9}N_{18}-E\right)}^{-1}[\left(N_{1}N_{18}-N_{10}\right)\Delta \theta_{G}+\left(N_{2}N_{18}-N_{11}\right)\Delta \omega \\ +\left(N_{3}N_{18}-N_{12}\right)\Delta P_{T}+\left(N_{4}N_{18}-N_{13}\right)\Delta k+\left(N_{5}N_{18}-N_{14}\right)\Delta P_{\mathrm{eW}1}\\ +\left(N_{6}N_{18}-N_{15}\right)\Delta P_{\mathrm{eW}2}+\left(N_{7}N_{18}-N_{16}\right)\Delta P_{\mathrm{eW}3}+\left(N_{8}N_{18}-N_{17}\right)] \end{array}\right.$$

Therefore, based on Equations (1), (47), and (49), the final system state equation for post-fault frequency response in high-penetration wind power grids is obtained:

(50) $$\left\{\begin{array}{l} \left[\begin{array}{c} \Delta \dot{\theta }\\ \Delta \dot{\omega }\\ \Delta {\dot{P}}_{T}\\ \Delta \dot{k}\\ \Delta {\dot{P}}_{\mathrm{eW}1}\\ \Delta {\dot{P}}_{\mathrm{eW}2}\\ \Delta {\dot{P}}_{\mathrm{eW}3} \end{array}\right]=\left[\begin{array}{ccccccc} 0 & \omega_{B}M_{1} & 0 & 0 & 0 & 0 & 0\\ -F_{1} & -M_{2}-F_{2} & E & 0 & -E_{1} & -E_{2} & -E_{3}\\ 0 & -M_{4} & -M_{5} & M_{3} & 0 & 0 & 0\\ 0 & -M_{7} & 0 & M_{6} & 0 & 0 & 0\\ 0 & 0 & 0 & 0 & M_{8} & 0 & 0\\ K_{1} & K_{2} & K_{3} & K_{4} & K_{5} & K_{6} & K_{7}\\ K_{9} & K_{10} & K_{11} & K_{12} & K_{13} & K_{14} & K_{15} \end{array}\right]\left[\begin{array}{c} \Delta \theta \\ \Delta \omega \\ \Delta P_{T}\\ \Delta k\\ \Delta P_{\mathrm{eW}1}\\ \Delta P_{\mathrm{eW}2}\\ \Delta P_{\mathrm{eW}3} \end{array}\right]+\left[\begin{array}{c} 0\\ F_{3}\\ 0\\ 0\\ -M_{9}\Delta v_{1}\\ K_{8}\\ K_{16} \end{array}\right]\\ \Delta \omega_{\mathrm{sys}}=\left[\begin{array}{ccccccc} 0 & K_{17} & 0 & 0 & 0 & 0 & 0 \end{array}\right]{\left[\begin{array}{ccccccc} \Delta \theta & \Delta \omega & \Delta P_{T} & \Delta k & \Delta P_{\mathrm{eW}1} & \Delta P_{\mathrm{eW}2} & \Delta P_{\mathrm{eW}3} \end{array}\right]}^{-1} \end{array}\right.$$

From this, the coefficient matrix is as follows:

$K_{i}=\left\{\begin{array}{l} {\left(N_{9}N_{18}-E\right)}^{-1}\left(N_{\left(9+i\right)}N_{9}-N_{i}\right)\;(i=1,\ldots ,8)\\ {\left(N_{9}N_{18}-E\right)}^{-1}\left(N_{\left(i-8\right)}N_{18}-N_{\left(i+1\right)}\right)\;(i=9,\ldots ,16) \end{array}\right.,K_{17}=\left[\begin{array}{ccc} M_{1} & \cdots & M_{m} \end{array}\right]/M_{T}$

For the convenience of expression, Formula (50) is expressed in the following form:

(51) $$\left\{\begin{array}{l} \dot{x}(t)=Ax(t)+b\\ \Delta \omega_{\mathrm{sys}}(t)=cx(t) \end{array}\right.$$

In the formula, A is the state matrix in the post-fault system state equation, b is the input component corresponding to the system state equation, and x(t) is the state variable vector of the system.

Based on linear system theory, solving the system state through Equation (51) yields the analytical model for predicting the post-fault dynamic response of the system inertial center frequency in high-penetration wind power grids:

(52) $$\Delta \omega_{\mathrm{sys}}(t)=c\sum_{i=1}^{r}(u_{i}w_{i})\left[X_{0}e^{\lambda_{i}t}+b\int_{0}^{t}e^{\lambda_{i}(t-\tau )}d\tau \right]=c\sum_{i=1}^{r}\frac{\left(u_{i}w_{i})b(1-e^{\lambda_{i}t}\right)}{-\lambda_{i}}$$

In the formula, r is the order of the system state equation; $\lambda_{i}$ is the eigenvalue of the system state matrix; $u_{i}$ is the right eigenvector corresponding to the eigenvalue; $w_{i}$ is the ir row of the inverse matrix of the right characteristic matrix; and $X_{0}$ is the initial state of the post-fault system state equation, with all elements being zero.

Therefore, when there is disturbance in the high-permeability grid of wind power, the required electromagnetic power of each generator end before disturbance is obtained through the wide-area measurement system. After disturbance, the electromagnetic power of each generator terminal, the load of each node, the voltage of each node (amplitude, phase angle) and the operating status parameters of each wind farm under different wind conditions can be obtained. Using the dynamic prediction model of the inertial center of the wind power grid shown in Formula (52), the frequency dynamic response characteristics of the wind power high-permeability power grid under the disturbance can be predicted.

Therefore, when a fault occurs in a high-penetration wind power grid, the required pre-fault electromagnetic power at each generator terminal, post-fault electromagnetic power at $t=0^{+}$, load data at each node, nodal voltages (magnitude and phase angle), and operating state parameters of wind farms under different wind conditions can be acquired via the wide-area measurement system. Based on the inertial center frequency dynamic prediction model, the post-fault frequency dynamic response characteristics of the high-penetration wind power grid under the fault can be accurately predicted. It should be noted that, in practical engineering applications, to ensure the accuracy of the prediction model, the obtained wide-area measurement data must undergo standard preprocessing processes such as data cleaning and bad data identification to ensure the quality of input data.

## 5. Simulation Analysis

### 5.1. Benchmark Power System Case

**Figure 2:**
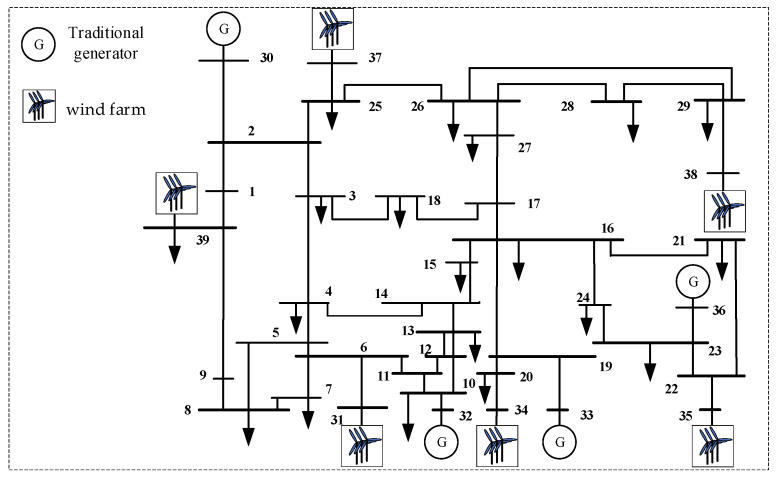
The Improved IEEE 39-node System.

To validate the correctness and effectiveness of the proposed method, the PSS/E simulation platform was utilized to modify the standard IEEE 39-bus system. Traditional generator units at Nodes 31, 34, 35, 37, 38, and 39 were replaced with equivalent wind farms, transforming the original grid into the high-penetration wind power system shown in Figure 2. The output of the wind farms was proportionally scaled relative to conventional units to adjust wind power penetration rates.

Time-domain simulation data from PSS/E under predefined fault scenarios were used to emulate the wide-area measurement system data required by the proposed method. The frequency response prediction model was implemented on the MATLAB R2023a platform. Key configurations included the following: All conventional units were equipped with governors capable of primary frequency regulation. All wind turbines provided frequency support during grid faults. Wind speed operating ranges were defined as follows—low wind speed: $0\leq v_{m}\leq 5$ m/s; medium wind speed: $5<v_{m}\leq 12$ m/s; high wind speed: $12<v_{m}\leq 16$ m/s. Cut-in and cut-out speeds were set at 3 m/s and 16 m/s, respectively. The rotor speed protection threshold was configured at 0.7 p.u., meaning units below this limit did not participate in frequency regulation.

Under different levels of wind speed conditions, the main parameters of the equivalent model of the WTGS are listed in Table 1 below.

For convenience of expression, in all the following cases, the proposed improved frequency response model is labeled as IFRM, the conventional frequency response model proposed in [7] is labeled as CFRM, and the time-domain simulation model of PSS/E is labeled as TDSM. It should be noted that, although the CFRM used for comparison in this paper also considers the aggregation of wind turbines participating in frequency regulation under low-, medium-, and high-wind-speed scenarios, it is essentially an equivalent model improved based on the traditional SFR model.

#### 5.1.1. Frequency Response Analysis at the Same Wind Speed

Validation under uniform wind speed conditions was conducted by maintaining the wind speed at all regional wind farms at 10.5 m/s with a wind power penetration rate of 50%. At t = 5 s, two distinct scenarios were simulated: a low-frequency fault scenario involving a short-circuit fault on the line between Nodes 17 and 18, resulting in 80% load shedding at Node 18, and a high-frequency disturbance scenario with a sudden 50% load increase at Node 15.

**Figure 3:**
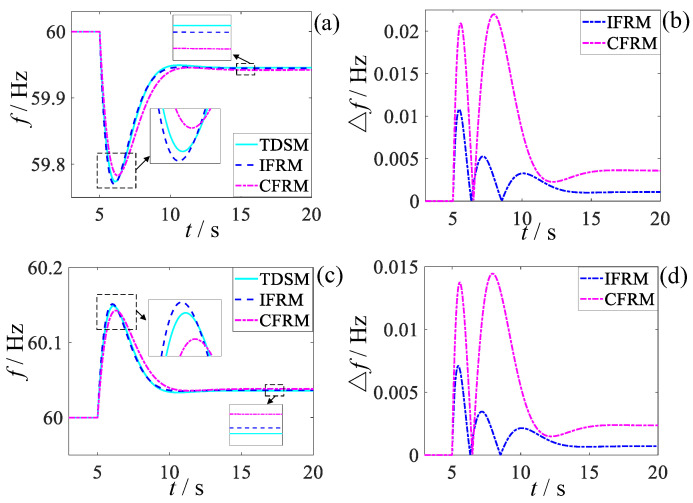
Comparison of frequency response prediction performance across different models: (**a**) Frequency response curves under low-frequency disturbances; (**b**) Prediction errors under low-frequency disturbances; (**c**) Frequency response curves under high-frequency disturbances; (**d**) Prediction errors under high-frequency disturbances.

For these operating conditions, pre-fault/steady-state and instantaneous post-fault/post-disturbance grid status data acquired from PSS/E served as inputs to the proposed IFRM model for predicting post-contingency frequency response characteristics. These predictions were compared against results generated by the CFRM model from existing literature and the TDSM model simulations from PSS/E, as illustrated in Figure 3.

**Figure 4:**
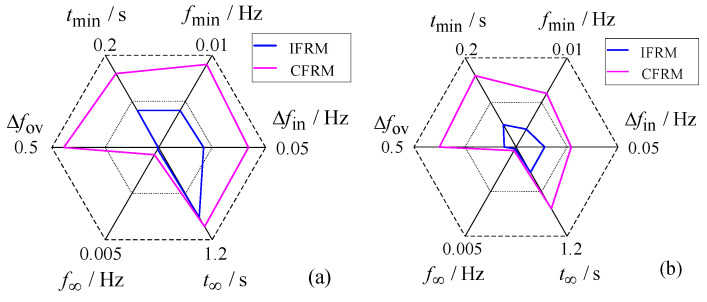
Comparison of prediction errors for key frequency response characteristics: (**a**) Under low-frequency disturbances; (**b**) Under high-frequency disturbances.

This paper selects the initial frequency change rate $\Delta f_{in}$, the lowest frequency $f_{\min}$, the lowest frequency arrival time $t_{\min}$, overmodulation $\Delta f_{\mathrm{ov}}$, steady-state frequency $f_{\infty }$ and steady-state frequency recovery time of the frequency response curve $t_{\infty }$ as the key frequency characteristic quantities. For different load disturbances, the key characteristic quantities and absolute error of the frequency prediction curve in the statistical Figure 3 are compared. The results are shown in Table 2 and Figure 4 below.

It can be seen from Figure 3 and Figure 4 and Table 2 that, although all wind farms have the same FM characteristics at the same wind speed, whether facing the low-frequency disturbance or high-frequency disturbance, the IFRM model mentioned in this paper has a better prediction effect than the CFRM model in the existing literature. For low-frequency disturbance, its lowest frequency and the steady-state frequency prediction accuracy can be improved by 55.56% and 33.33%, respectively, and for high-frequency disturbances, the lowest frequency and steady-state frequency prediction accuracy can be improved by 66.67% and 50%, respectively. In the same-medium-wind-speed state, although the IFRM model and the CFRM model can both consider the impact of the integrated inertial control of the fan on the frequency response, the IFRM model can also take into account the network loss of the power grid, changes in load characteristics, and the differences in FM characteristics of each synchronous generator set at the same time. For the impact on frequency, its frequency response prediction accuracy is higher and more advantageous than that of traditional CFRM models.

As evidenced by Figure 3 and Figure 4 and Table 2, the proposed IFRM model—leveraging wide-area measurement data—demonstrates significantly superior prediction accuracy over the CFRM model when confronting both low-frequency faults and high-frequency disturbances, despite all wind farms operating under identical medium wind speeds with consistent frequency regulation characteristics. Specifically for low-frequency faults, prediction accuracy improvements reach 55.56% for the frequency nadir and 33.33% for steady-state frequency. For high-frequency disturbances, accuracy enhancements attain 66.67% for $f_{\min}$ and 50% for $f_{\infty }$. This precision advantage stems from the IFRM model’s comprehensive utilization of granular grid-state information provided by wide-area measurements, which simultaneously accounts for network losses, load-dynamic characteristic variations, and frequency regulation characteristic disparities among synchronous units in shaping post-fault frequency response. In contrast, the CFRM model merely considers the aggregated frequency regulation effect of wind power.

#### 5.1.2. The Frequency Response Analysis Under Power Shortage and Changing Wind Speed

Validation under multi-wind-speed-coexistence conditions was conducted with the system-wide wind power penetration rate set at 50%. The fault scenario involved a short-circuit between Nodes 17 and 18 occurring at time t equals 5 s, resulting in 100% load shedding at Node 18. Three distinct operational scenarios were simulated for comparative analysis: Scenario A configured wind farms connected to Nodes 37 and 39 operating under low wind speed at 3 m per second, those at Nodes 31 and 34 under medium wind speed at 8 m per second, and those at Nodes 35 and 38 under high wind speed at 13 m per second. Scenario B maintained low-wind-speed operation at Nodes 37 and 39 despite a 2 m per second increase, reaching 5 m per second in total, while wind farms at Nodes 35 and 38 entered medium-wind-speed operation after a 2 m per second decrease, reaching 11 m per second in total, with other conditions mirroring Scenario A. Scenario C transitioned wind farms at Nodes 37 and 39 to medium-wind-speed operation after a 4 m per second increase, reaching 7.5 m per second in total, while those at Nodes 35 and 38 entered medium-wind-speed operation following a 2 m per second decrease, reaching 11 m per second in total, retaining other conditions identical to Scenario A.

**Figure 5:**
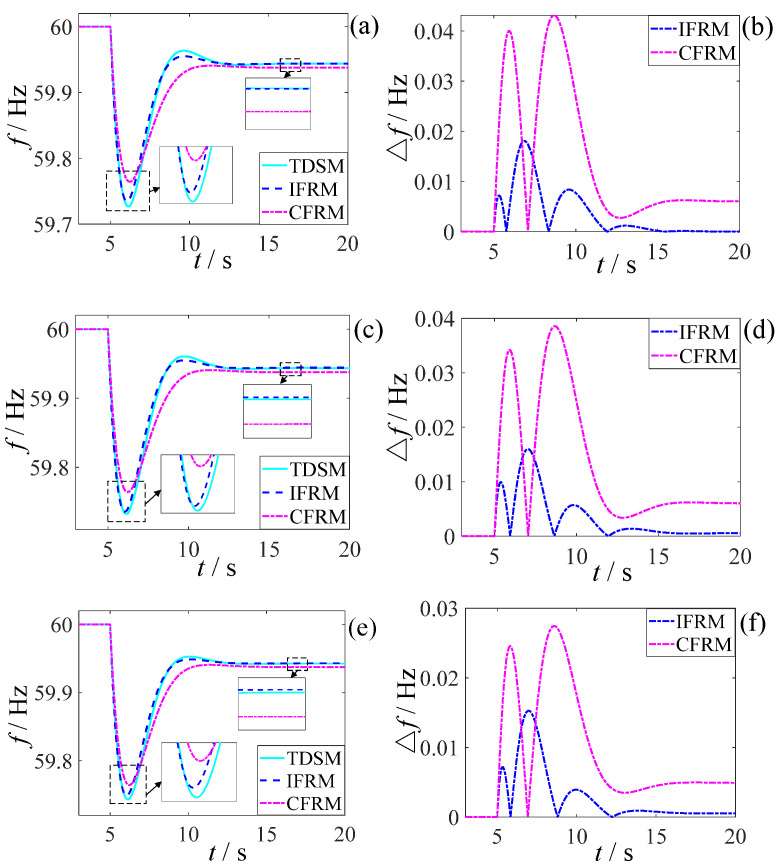
Comparison of frequency response prediction performance across different scenarios. (**a**) Frequency response curves in Scenario A; (**b**) Prediction errors in Scenario A; (**c**) Frequency response curves in Scenario B; (**d**) Prediction errors in Scenario B; (**e**) Frequency response curves in Scenario C; (**f**) Prediction errors in Scenario C.

For these scenarios, wide-area measurement data before and after faults were input into the IFRM model for prediction. The CFRM model, being limited to single-wind-speed input, uniformly adopted the average wind speed across all farms at 8 m per second while assuming all turbines operated under medium-wind-speed conditions. Comparative results of IFRM, CFRM, and TDSM benchmark simulations are presented in Figure 5.

**Figure 6:**
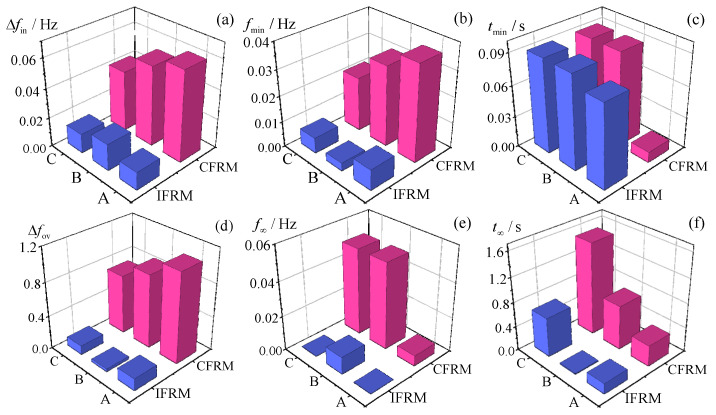
Comparison of prediction errors for key frequency response characteristics in different scenarios. (**a**) Δ*f_in_*; (**b**) *f_min_*; (**c**) *t_min_*; (**d**) Δ*f_ov_*; (**e**) *f_∞_*; (**f**) *t_∞_*.

To further highlight prediction discrepancies between IFRM and CFRM across Scenarios A, B, and C, absolute errors for key frequency response characteristics were analyzed as shown in Figure 6. Numerical comparisons of these characteristics are detailed in the subsequent section.

Analysis of Figure 5 reveals that across all three scenarios the prediction results from the proposed IFRM model demonstrate closer alignment with TDSM time-domain simulation outcomes compared to the existing CFRM model, exhibiting superior overall prediction accuracy. The CFRM model generated identical frequency response curves under all three scenarios due to its invariant wind speed input configuration and identical grid disturbance conditions. Simultaneously, the comparison of key frequency response characteristic errors in Figure 6 indicates that CFRM prediction errors decrease significantly as wind turbines approach medium-speed operating conditions, whereas the IFRM model maintains consistent error levels regardless of operational transitions. Corresponding to Scenarios A, B, and C, the accuracy improvement ratios of the IFRM model relative to the CFRM model for key frequency characteristics are quantified as detailed in Table 3.

As indicated in Table 3, CFRM prediction errors escalate significantly as more wind farms deviate from medium-speed operation, reducing overall accuracy. Conversely, the IFRM model’s advantage becomes more pronounced. This stems from IFRM’s ability to account for frequency regulation characteristic differences arising from spatial wind speed variations among wind farms. CFRM, being limited to aggregated virtual inertia control under a single wind speed, fails to capture real-time regulation characteristic changes despite unchanged average wind speed. Consequently, CFRM exhibits substantial deviations from time-domain simulations, with errors amplifying as wind speed diversity and operational dispersion increase across the grid. This case conclusively validates IFRM’s capability to accurately reflect the impact of spatially heterogeneous wind farm frequency regulation characteristics during local wind speed variations.

#### 5.1.3. The Analysis of the Frequency Response Model Under Different Wind Power Penetration

**Figure 7:**
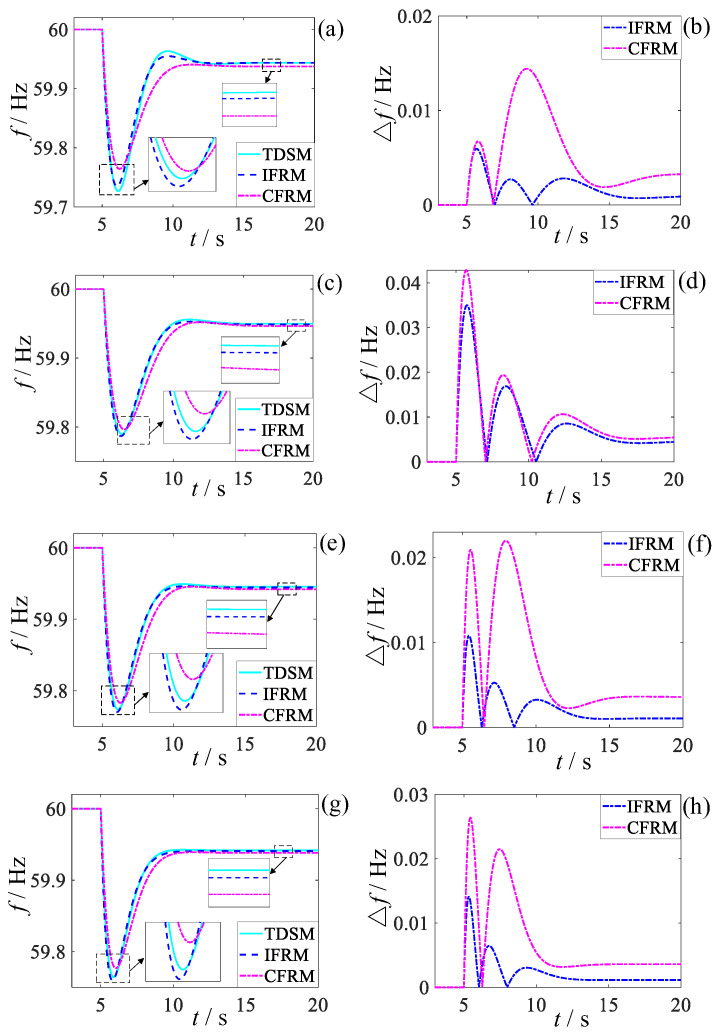
Comparison of frequency response prediction performance under different wind power penetration levels. (**a**) Frequency response curves (20%); (**b**) Prediction errors (20%); (**c**) Frequency response curves (40%); (**d**) Prediction errors (40%); (**e**) Frequency response curves (60%); (**f**) Prediction errors (60%); (**g**) Frequency response curves (80%); (**h**) Prediction errors (80%).

The frequency response analysis model proposed in this paper is used for high-penetration wind power grid frequency response, so it is necessary to prove the accuracy of the model under different penetration rates. The system conditions of this case are set to be the same as the low-frequency disturbance scenario in Case 1, and merely the wind power penetration rate of the system is changed to 20%, 40%, 60%, and 80%, respectively. When t = 5s, the load experiences an 80% power shortage. The frequency and absolute error response results of IFRM and CFRM are compared with those of TDSM, as shown in Figure 7.

**Figure 8:**
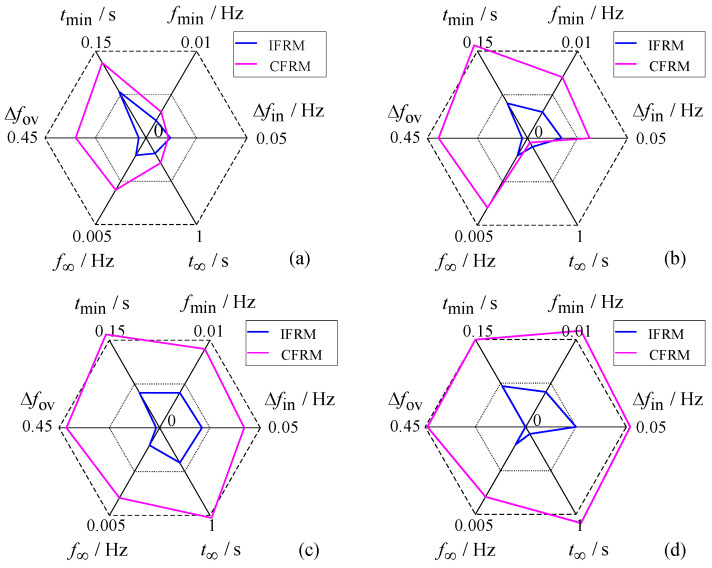
Comparison of prediction errors for key frequency response characteristics under different wind power penetration levels. (**a**) 20%; (**b**) 40%; (**c**) 60%; (**d**) 80%.

In order to highlight the applicability of the proposed model, the absolute errors of the frequency key characteristic obtained by the two models under different wind power penetration are compared. The results are shown in Figure 8 below:

Comparative analysis of the penetration rate impacts in Figure 7 and Figure 8 reveals distinct patterns in post-fault frequency prediction performance between the IFRM and CFRM models as wind power penetration varies. At penetration rates of 20% and 40%, the data-driven IFRM model maintains relatively balanced prediction errors, achieving an average accuracy improvement of 42.95% across key characteristics under low-penetration conditions. In contrast, CFRM errors exhibit a pronounced upward trend with increasing penetration. When wind power penetration reaches 60% and 80%, the CFRM model suffers severe error deterioration due to its overreliance on single-wind-speed assumptions and neglect of network dynamic coupling. Conversely, leveraging the precise system-wide state perception enabled by wide-area measurements, the IFRM model sustains stable prediction errors at low levels, delivering average accuracy improvements of 63.47% and 62.625% at 60% and 80% penetration, respectively. These results not only validate IFRM’s strong applicability in high-penetration scenarios but also expose a fundamental limitation of conventional CFRM: its inability to accommodate the dramatic increase in system complexity resulting from elevated wind penetration during post-fault frequency stability analysis. As wind penetration increases, the accuracy advantage of the proposed data-driven method becomes progressively more pronounced.

### 5.2. Real-World Power System Case

**Figure 9:**
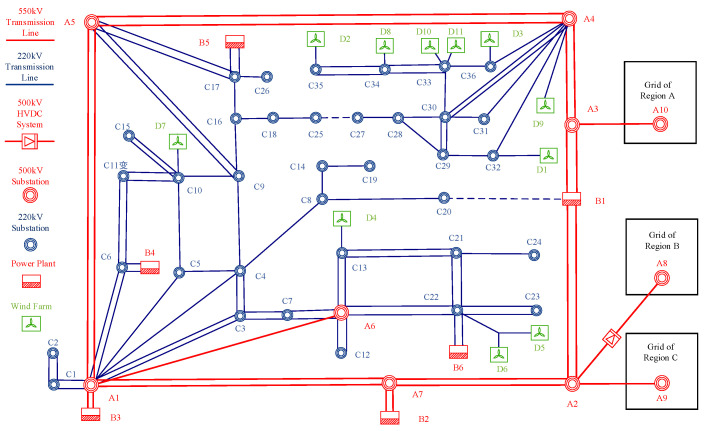
An Actual Renewable Energy Sending-End Grid.

To further validate the engineering applicability of the proposed method, this case study performs frequency response analysis on an actual renewable energy sending-end grid in Northern China. The grid comprises 62 buses, with its topology illustrated in Figure 9.

The system includes five thermal units (B1–B5) and 11 wind farms (D1–D11). The wind speeds at each farm are 14 m/s, 13 m/s, 15 m/s, 3 m/s, 5 m/s, 2 m/s, 8 m/s, 5 m/s, 7 m/s, 10 m/s, and 12 m/s, respectively, with a renewable penetration level of 46.7%. The actual grid model is built in PSS/E.

**Figure 10:**
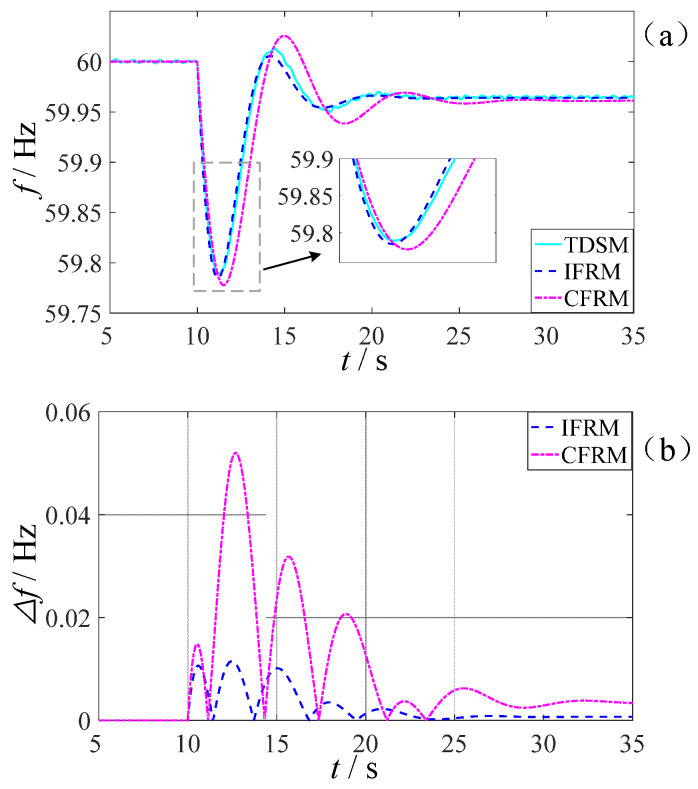
Comparison of frequency response prediction performance. (**a**) Frequency response curves of IFRM, CFRM, and TDSM results; (**b**) Prediction errors of IFRM and CFRM relative to TDSM reference.

A 20% sudden load increase is applied at Bus C4 when t = 10 s. Wide-area measurement system data at t = 10.02 s (post-disturbance) are used as the initial input for the proposed model. The frequency response predictions of the proposed IFRM model and conventional CFRM model are compared against TDSM results in Figure 10.

A statistical comparison of the key frequency characteristics in Figure 10 is presented in Table 4.

According to the actual grid simulation results summarized in Table 4, the proposed IFRM model maintains excellent performance in practical complex power grids, further validating its engineering applicability. In terms of dynamic characteristic prediction, the accuracy of IFRM in predicting the time to frequency nadir is improved by 5.07% compared to CFRM, showing closer alignment with detailed PSS/E simulation results. For steady-state process characterization, the settling time of IFRM is reduced by 25.12% relative to CFRM, significantly enhancing the accuracy of dynamic process simulation. Notably, IFRM predictions for the critical initial rate-of-frequency-decline metric show strong consistency with PSS/E measured data, with a 38.2% reduction in error compared to CFRM. These results fully demonstrate that the proposed method effectively adapts to complex operational environments in actual power grids by integrating wide-area measurement information, thereby providing more reliable frequency security early-warning support for dispatch operations.

## 6. Conclusions

To address the need for accurate prediction of fault-induced frequency response characteristics in power grids with high wind power penetration, this study proposes a data-driven linearized frequency dynamic response prediction method based on wide-area measurement data. By integrating panoramic power grid state information, the established model overcomes the limitations of existing methods. On the basis of considering network losses, dynamic load characteristics, and differences in the regulation of synchronous units, it innovatively incorporates the actual wind speed conditions of regional wind farms, establishes a refined frequency regulation model for wind turbine units under low-/medium-/high-wind-speed scenarios, and reveals the complex coupling mechanism between wind power clusters and system frequency response.

Simulation results show that the prediction accuracy of the proposed method is significantly superior to that of traditional aggregated models: In the uniform wind speed scenario, the prediction accuracy of the frequency nadir and steady-state frequency are improved by 55.56–66.67% and 33.33–50%, respectively; in the scenario with coexisting multiple wind speeds, the prediction accuracy of key characteristic parameters is improved by 20–100%, and the advantage becomes more prominent as the wind speed dispersion increases; within the wind power penetration range of 20–80%, the average prediction accuracy is improved by 42.95–63.47%. In the real-world power grid case, the prediction accuracy of the time to frequency nadir is improved by 5.07%, the steady-state settling time is shortened by 25.12%, and the prediction error of the initial rate of frequency decline is reduced by 38.2%.

The above quantitative results verify the effectiveness and engineering applicability of the proposed method in complex power grid environments. Future research will focus on exploring the impact of WAMS data quality issues on frequency prediction and the corresponding compensation methods, and advance the construction of a hybrid frequency prediction framework integrating data-driven and physical models to further enhance the robustness and accuracy of the model.

## Figures and Tables

**Table 1:** Simulation parameters of WTGS.

Model	Low Wind	Medium Wind	High Wind
*H_W_*	5.2	5.8	6.2
*w*	0.765	0.798	0.8054
*K_w_*	0.1	0.16	0.18
*K_P_*	0.68	0.68	0.73
*K_c_*	0.0771	0.0688	0.0722
*R_v_*	0.05	0.05	0.05
*K_β_*	/	/	−0.1015
*D_b_*	/	/	550

**Table 2:** Comparison results of frequency characteristic values under different disturbances.

Fault Type	Model	Frequency Response Characteristics					
		Δ*f*_in_/Hz	*f*_min_/Hz	*t*_min_/s	Δ*f*_ov_	*f*_∞_/Hz	*t*_∞_/s
Low-frequency fault	IFRM	0.037	59.770	6.02	318.182%	59.945	15.41
	CFRM	0.307	59.783	6.26	274.13%	59.942	17.35
	TDSM	0.349	59.774	6.10	318.519%	59.946	16.32
High-frequency disturbance	IFRM	0.243	60.151	6.02	308.108%	60.037	14.63
	CFRM	0.202	60.143	6.23	276.316%	60.038	14.97
	TDSM	0.229	60.149	6.07	313.889%	60.036	14.14

**Table 3:** Comparison results of frequency characteristic values under different scene types.

Scene Type	The Rate of Improvement of Key Feature Prediction Accuracy/(%)					
	Δ*f*_in_	*f*_min_	*t*_min_	Δ*f*_ov_	*f*_∞_	*t*_∞_
A	80.33	78.38	20	86.79	100	50
B	66.67	90.32	0	96.59	80	98.61
C	68.29	71.43	0	85.71	100	58.28

**Table 4:** Frequency response characteristics.

Model	Rate of Change in Frequency					
	Δ*f*_in_/Hz	*f*_min_/Hz	*t*_min_/s	Δ*f*_ov_	*f*_∞_/Hz	*t*_∞_/s
IFRM	0.287	59.785	6.18	326.147%	59.963	20.5
CFRM	0.246	59.776	6.51	446.134%	59.960	29.7
TDSM	0.273	59.789	6.22	328.054%	59.964	21.8

## Data Availability

The data presented in this study are available on request from the corresponding author.

## Abbreviations

The following abbreviations are used in this manuscript:

CFRM	Conventional Frequency Response Model
DOF	Degree of Freedom
FM	Frequency Modulation
HPP	High-Penetration Power (grid)
IFRM	Improved Frequency Response Model
LD	Load Disturbance
MPPT	Maximum Power Point Tracking
P-ROFR	Segmented Reduced-Order Frequency Response
SFR	System Frequency Response
TDSM	Time-Domain Simulation Model
WAMS	Wide-Area Measurement System
WTGS	Wind Turbine Generator System

## Appendix A

$$\begin{array}{l} N_{1}={\left(E+M_{11}E_{2}\right)}^{-1}\left[M_{11}\left(M_{2}+F_{2}\right)F_{1}-M_{12}F_{1}\right]\\ N_{2}={\left(E+M_{11}E_{2}\right)}^{-1}\left[M_{11}{\left(M_{2}+F_{2}\right)}^{2}-M_{12}\left(M_{2}+F_{2}\right)-M_{11}M_{4}-\omega_{B}M_{11}F_{1}M_{1}+M_{13}\right]\\ N_{3}={\left(E+M_{11}E_{2}\right)}^{-1}\left[M_{11}M_{2}+M_{11}F_{2}+M_{12}-M_{11}M_{5}\right]\\ N_{4}={\left(E+M_{11}E_{2}\right)}^{-1}M_{11}M_{3}\\ N_{5}={\left(E+M_{11}E_{2}\right)}^{-1}\left[M_{11}\left(M_{2}+F_{2}\right)E_{1}-M_{12}E_{1}-M_{11}E_{1}M_{8}\right]\\ N_{6}={\left(E+M_{11}E_{2}\right)}^{-1}\left[M_{11}\left(M_{2}+F_{2}\right)E_{2}-M_{12}E_{2}-M_{10}\right]\\ N_{7}={\left(E+M_{11}E_{2}\right)}^{-1}\left[M_{11}\left(M_{2}+F_{2}\right)E_{3}-M_{12}E_{3}\right]\\ N_{8}={\left(E+M_{11}E_{2}\right)}^{-1}\left[\left(M_{14}+M_{11}E_{1}M_{9}\right)\Delta v+M_{12}F_{3}-M_{11}\left(M_{2}+F_{2}\right)F_{3}\right]\\ N_{9}={\left(E+M_{11}E_{2}\right)}^{-1}M_{11}E_{3}\\ N_{10}={\left(E+M_{16}E_{2}\right)}^{-1}\left[M_{16}\left(M_{2}+F_{2}\right)F_{1}-M_{17}F_{1}\right]\\ N_{11}={\left(E+M_{16}E_{2}\right)}^{-1}\left[M_{16}{\left(M_{2}+F_{2}\right)}^{2}-M_{17}\left(M_{2}+F_{2}\right)-M_{16}M_{4}-\omega_{B}M_{16}F_{1}M_{1}+M_{18}\right]\\ N_{12}={\left(E+M_{16}E_{2}\right)}^{-1}\left[M_{16}M_{2}+M_{16}F_{2}+M_{17}-M_{16}M_{5}\right]\\ N_{13}={\left(E+M_{16}E_{2}\right)}^{-1}M_{16}M_{3}\\ N_{14}={\left(E+M_{16}E_{2}\right)}^{-1}\left[M_{16}\left(M_{2}+F_{2}\right)E_{1}-M_{17}E_{1}-M_{16}E_{1}M_{8}\right]\\ N_{15}={\left(E+M_{16}E_{2}\right)}^{-1}\left[M_{16}\left(M_{2}+F_{2}\right)E_{3}-M_{17}E_{3}\right]\\ N_{16}={\left(E+M_{16}E_{2}\right)}^{-1}\left[M_{16}\left(M_{2}+F_{2}\right)E_{2}-M_{17}E_{2}-M_{15}\right]\\ N_{17}={\left(E+M_{16}E_{2}\right)}^{-1}\left[\left(M_{19}+M_{16}E_{1}M_{9}\right)\Delta v+M_{17}F_{3}-M_{16}\left(M_{2}+F_{2}\right)F_{3}\right]\\ N_{18}={\left(E+M_{16}E_{2}\right)}^{-1}M_{16}E_{3} \end{array}$$

$$\begin{array}{l} M_{1}=\left[\begin{array}{ccccccc} 1 & & & \overset{k\text{-}\mathrm{th}\;\mathrm{column}}{\overbrace{-1}} & & & \\ & \ddots & & \vdots & & 0 & \\ & & 1 & -1 & & & \\ & & & -1 & 1 & & \\ & 0 & & \vdots & & \ddots & \\ & & & -1 & & & 1 \end{array}\right]\\ M_{2}=\mathrm{diag}\left\{D_{ti}\right\}\\ M_{3}=\mathrm{diag}\left\{\left(T_{1i}-T_{2i})/(T_{1i}T_{3i}\right)\right\}\\ M_{4}=\mathrm{diag}\left\{T_{2i}/(R_{i}T_{1i}T_{3i})\right\}\\ M_{5}=\mathrm{diag}\left\{1/T_{3i}\right\}\\ M_{6}=\mathrm{diag}\left\{1/T_{1i}\right\}\\ M_{7}=\mathrm{diag}\left\{1/(R_{i}T_{1i})\right\}\\ M_{8}=\mathrm{diag}\left\{b_{li}/c_{li}\right\}\\ M_{9}=\mathrm{diag}\left\{a_{li}/c_{li}\right\}\\ M_{10}=\mathrm{diag}\left\{1/b_{mi}\right\}\\ M_{11}=\mathrm{diag}\left\{c_{mi}/b_{mi}\right\}\\ M_{12}=\mathrm{diag}\left\{d_{mi}/b_{mi}\right\}\\ M_{13}=\mathrm{diag}\left\{e_{mi}/b_{mi}\right\}\\ M_{14}=\mathrm{diag}\left\{a_{mi}/b_{mi}\right\}\\ M_{15}=\mathrm{diag}\left\{1/b_{hi}\right\}\\ M_{16}=\mathrm{diag}\left\{c_{hi}/b_{hi}\right\}\\ M_{17}=\mathrm{diag}\left\{d_{hi}/b_{hi}\right\}\\ M_{18}=\mathrm{diag}\left\{e_{hi}/b_{hi}\right\}\\ M_{19}=\mathrm{diag}\left\{a_{hi}/b_{hi}\right\} \end{array}$$
